# Associations of Toll-like Receptor Gene Polymorphisms with NETosis Activity as Prognostic Criteria for the Severity of Pneumonia

**DOI:** 10.17691/stm2021.13.3.06

**Published:** 2021-06-28

**Authors:** M.A. Karnaushkina, A.S. Guryev, K.O. Mironov, E.A. Dunaeva, V.I. Korchagin, O.Yu. Bobkova, I.S. Vasilyeva, D.V. Kassina, M.M. Litvinova

**Affiliations:** Professor, Department of Internal Diseases with a Course of Cardiology and Functional Diagnostics named after Academician V.S. Moiseev; Peoples’ Friendship University of Russia, 6 Miklukho-Maklaya St., Moscow, 117198, Russia; Senior Researcher, Research Laboratory; Moscow Regional Research Clinical Institute named after M.F. Vladimirsky, 61/2–1 Schepkina St., Moscow, 129110, Russia; Head of the Research Group for the Development of New Methods for Identifying Genetic Polymorphisms; Central Research Institute of Epidemiology of the Federal Service on Consumer Rights Protection and Human Wellbeing (Rospotrebnadzor), 3а Novogireevskaya St., Moscow, 111123, Russia; Researcher, Research Group for the Development of New Methods for Identifying Genetic Polymorphisms; Central Research Institute of Epidemiology of the Federal Service on Consumer Rights Protection and Human Wellbeing (Rospotrebnadzor), 3а Novogireevskaya St., Moscow, 111123, Russia; Researcher, Research Group for the Development of New Methods for Identifying Genetic Polymorphisms; Central Research Institute of Epidemiology of the Federal Service on Consumer Rights Protection and Human Wellbeing (Rospotrebnadzor), 3а Novogireevskaya St., Moscow, 111123, Russia; PhD Student, Department of Hospital Therapy No.2; I.M. Sechenov First Moscow State Medical University (Sechenov University), 8/2 Trubetskaya St., Moscow, 119991, Russia; Assistant, Department of Hospital Therapy No.2; I.M. Sechenov First Moscow State Medical University (Sechenov University), 8/2 Trubetskaya St., Moscow, 119991, Russia; Researcher, Research Laboratory; Moscow Regional Research Clinical Institute named after M.F. Vladimirsky, 61/2–1 Schepkina St., Moscow, 129110, Russia; Associate Professor, Department of Medical Genetics; I.M. Sechenov First Moscow State Medical University (Sechenov University), 8/2 Trubetskaya St., Moscow, 119991, Russia; Geneticist, Center for Personalized Medicine; Moscow Clinical Scientific Center named after A.S. Loginov, Moscow Healthcare Department, 86 Shosse Entuziastov, Moscow, 111123, Russia

**Keywords:** gene polymorphism, *TLR1*, *TLR2*, *TLR4*, neutrophilic extracellular traps, pneumonia

## Abstract

**The aim of the study** was to determine the molecular genetic prognostic criteria for the severity of the course pneumonia based on the analysis of the association of genetic polymorphism in toll-like receptors with the severity of NETosis.

**Materials and Methods:**

The study included 38 patients with the main diagnosis of community-acquired pneumonia with a severe course. All the patients underwent standard clinical laboratory examinations, computed tomography of the thoracic organs, microbiological examination of blood and tracheobronchial aspirate. The level of neutrophilic extracellular traps (NETs) in blood smears was determined on the 1^st^–2^nd^ and 5^th^–7^th^ days of hospitalization. Genotyping of rs5743551 (*TLR1*), rs5743708 (*TLR2*), and rs4986790 (*TLR4*) polymorphic loci was performed by pyrosequencing.

**Results:**

The level of NETs on the 1^st^ day of admission was statistically significantly lower in heterozygous and homozygous carriers of rs4986790 (*TLR4*) polymorphism (AG and GG genotypes) compared with patients with the wild-type genotype (AA genotype) (p<0.05). When comparing the number of NETs with genotypes for rs5743708 (*TLR2*) and rs5743551 (*TLR1*) polymorphisms, no statistically significant correlation was found (p>0.05). The study of the NET level in dynamics demonstrated a decrease in the NETosis activity of neutrophils during the first week of hospitalization (p<0.05). The presence of the G allele in the patient’s genotype for rs5743551 (*TLR1*) polymorphism increases the risk of a poor outcome of the disease (p<0.0001) (OR=20.3; 95% CI (4.3–135.0)).

**Conclusion:**

The obtained data suggest that level of NETs is a marker of the activity of neutrophils which are closely related to the studied genetic polymorphisms, and affects the prognosis of the pneumonia outcome.

## Introduction

Pneumonia is an acute infectious disease characterized by focal lesions of the respiratory portions of the lungs with interalveolar exudation. At the moment, pneumonia is one of the main causes of death in the world, and it accounts for 41.5% in the structure of mortality from respiratory diseases [1, 2]. Identification of genetic factors associated with severe and complicated pneumonia may contribute to the development of new approaches to the treatment of this disease and identification of early predictors of its adverse outcomes.

In 2004, a new function of neutrophils was described — the formation of neutrophilic extracellular traps (NETs), focused on the extracellular suppression of the activity of pathogens. NETs are DNA strands with adsorbed antimicrobial factors of neutrophil granules on them [3]. This antimicrobial defense mechanism has been termed “NETosis” [4]. Neutrophils massively undergo NETosis in infectious, non-infectious, and autoimmune diseases. In infectious diseases, a significant part of active granulocytes migrates to the surface of the mucous membranes, including the respiratory tract, where they exercise their protective functions [5–7]. In this case, NETs and activated neutrophils are found not only in mucosal secretions but also in the blood [7–9]. After interacting with NETs, most of the microorganisms die affected by the bactericidal substances that make up their composition. A variant of NETosis has also been described, in which, after the release of NETs, a neutrophil remains alive and continues to perform its functions [6].

However, some microorganisms can survive due to different mechanisms of antibacterial protection, which leads to the progression of the disease [9]. In this regard, the study of the mechanisms of the formation and regulation of neutrophil activity and NET formation is an urgent clinical task.

Since the activation of innate immunity begins with the phase of antigenic structure recognition, at present, special attention is paid to the study of toll-like receptors expressed by the cells of the immune system, including neutrophils. Since NETs can be formed only by activated neutrophils [4–6], and their activation occurs through toll-like receptors, the extracellular domains of which interact with the microorganism, it is of great importance to study the role of gene polymorphism in this group of receptors.

According to the literature [10, 11], rs5743708 (*TLR2* gene) and rs4986790 (*TLR4* gene) polymorphic loci are the most studied in patients with community-acquired pneumonia.

**The aim of the study** was to determine the molecular genetic prognostic criteria for the severity of pneumonia based on the analysis of the association of genetic polymorphisms of toll-like receptors (rs5743551 (*TLR1*), rs5743708 (*TLR2*), and rs4986790 (*TLR4*) loci) with the severity of NETosis.

## Materials and Methods

In the period from 2018 to 2019, a prospective study was planned and carried out on the basis of the City Clinical Hospital named after S.S. Yudin of the Moscow Healthcare Department (Russia) and the Central Research Institute of Epidemiology of Rospotrebnadzor (Moscow, Russia).

The criteria for patient recruiting in the study were the following: patients aged from 40 to 70 years; diagnosis of pneumonia confirmed by clinical and laboratory examination and CT scan of the thoracic organs; accordance with the criteria for community-acquired pneumonia; patient hospitalization in the ICU upon admission; absence of oncological diseases, HIV, B and C hepatitis, blood diseases, systemic connective tissue diseases, vasculitis, chronic severe bronchopulmonary pathology, and occupational diseases; no therapy with immunosuppressive drugs and systemic glucocorticoids.

Forty patients with community-acquired pneumonia who met the inclusion criteria were randomly selected from the patients admitted to the ICU of the City Clinical Hospital named after S.S. Yudin.

In compliance with the clinical guidelines for the diagnosis, treatment, and prevention of community-acquired pneumonia in adults [12], standard clinical and laboratory studies, CT of the chest, microbiological blood and tracheobronchial aspirate testing for aerobic and anaerobic flora were performed. The severity of the patient’s condition was assessed using the validated PORT and SOFA scales [13, 14].

The study included 38 out of 40 patients who underwent a complete examination (mean age — 44 (37–58) years), 63% were men, 37% were women.

**Figure 1:**
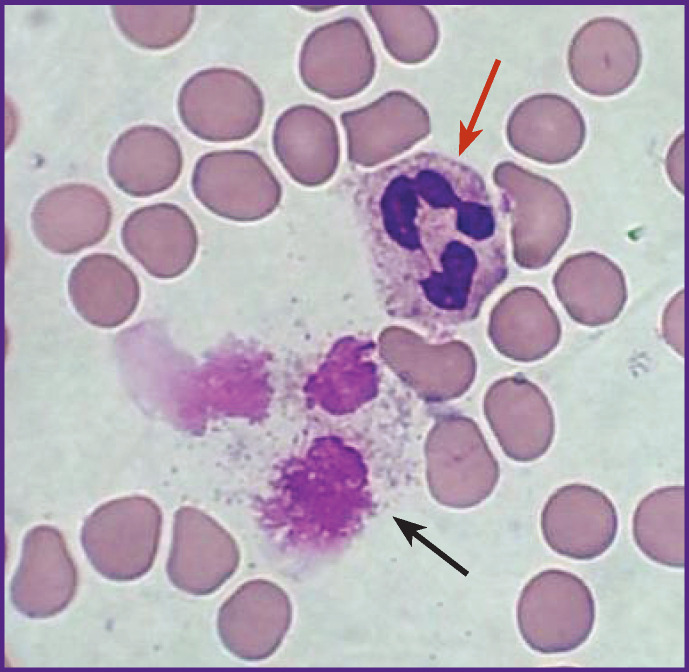
Neutrophil (*a red arrow*) and neutrophil extracellular trap (*a black arrow*), visualized in a “monolayer”-type blood smear, stained according to Romanowsky–Giemsa; ×5000

The NET level in venous blood samples was determined on the 1^st^–2^nd^ (first visit) and 5^th^–7^th^ (second visit) days of the patient’s hospitalization. Venous blood was taken in tubes with ethylenediaminetetraacetic acid (EDTA). Within an hour, the samples were used to prepare standardized “monolayer”-type smears, which were then stained according to Romanowsky–Giemsa and studied on a MECOS-C2 automated microscopy system (MECOS, Russia) in accordance with the original authors’ method [15] (Figure 1). For a low NET level, a value less than or equal to 12% in a smear was taken, for a high level — more than 12%.

A molecular genetic study was carried out on the basis of the Central Research Institute of Epidemiology of Rospotrebnadzor. Genotyping of rs5743551 (*TLR1*), rs5743708 (*TLR2*), and rs4986790 (*TLR4*) polymorphic loci was performed using reagents for DNA isolation (RIBO-prep), amplification and sample preparation (PIRO-prep) produced by the Central Research Institute of Epidemiology of Rospotrebnadzor (AmpliSens, Russia) by pyrosequencing using the PyroMark Q96 reagents and the PyroMark Q24 genetic analysis system (QIAGEN, Germany) [16].

All the procedures performed in the human studies were in compliance with the ethical standards of the National Research Ethics Committee and the Declaration of Helsinki (2013) or comparable ethical standards. Informed voluntary consent was obtained from each participant.

### Statistical analysis

The statistical data processing was performed using the Statistica 10.0 software. The Shapiro–Wilk W-test was used to determine the nature of the data distribution. The value differences between the groups were assessed using nonparametric tests — the Mann–Whitney U test and the Kruskal–Wallis rank test. When comparing the frequencies of the features in the groups, Fisher’s exact test was used. The differences were considered statistically significant at p<0.05. To analyze the association of the alleles/genotypes, the odds ratio (OR) was calculated.

## Results

The subjects were distributed as follows: the patients with pneumonia who did not require vasopressor support (n=31); the patients with pneumonia requiring vasopressor support (n=7). The study participants were stratified by the risk of adverse outcome (PORT scale); the genotype with respect to rs5743551 (*TLR1*), rs5743708 (*TLR2*), and rs4986790 (*TLR4*) polymorphisms; level of NETs; the outcome of the disease and the nature of the pathogen.

According to the PORT scale, 29 patients (76%) belonged to the 3^rd^ class, 9 patients — to the 4^th^ class. Patients of the 1^st^ and 2^nd^ classes who did not have the risk of an adverse outcome and were not treated in the ICU did not participate in the study.

When conducting a comparative intergroup analysis of the patients in the groups stratified by the presence and absence of complications, disease outcome, leukocyte and C-reactive protein (CRP) levels, no statistically significant differences in clinical and functional parameters and the level of NETs in blood smears were obtained.

At the next stage of the study, a hypothesis was suggested that the level of formed NETs depends not only on the clinical and laboratory characteristics of the course of pneumonia but also on the genetically determined activity of neutrophils. To confirm this hypothesis, on the basis of the results of a literature review and Online Mendelian Inheritance in Men (OMIM) genomic databases (https://omim.org/); ClinVar (https://www.ncbi.nlm.nih.gov/clinvar/); SNPedia (https://snpedia.com/); dbSNP (https://www.ncbi.nlm.nih.gov/snp/), three genetic polymorphisms of toll-like receptors were selected: rs5743551 (*TLR1*), rs5743708 (*TLR2*), and rs4986790 (*TLR4*) [11, 17, 18].

**Figure 2:**
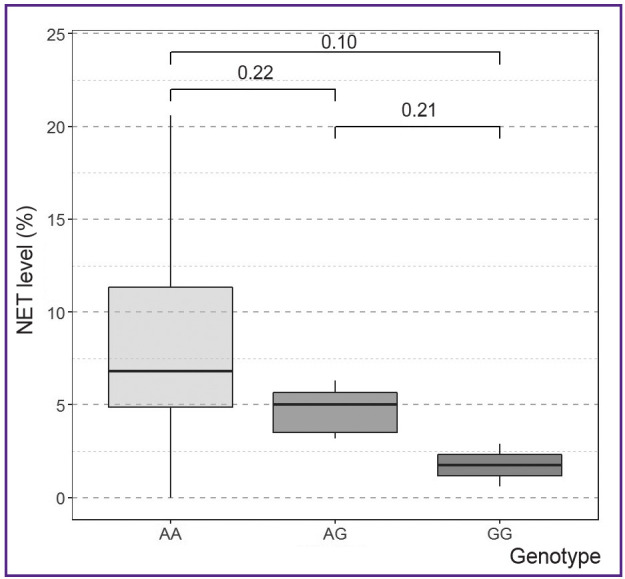
Dependence of the NET level on the genotype of the rs4986790 (*TLR4*) locus at the first blood sampling The p-values of the Mann–Whitney intergroup differences test with Bonferroni correction are given

When analyzing the association of the level of NETs during the first blood sampling with the genotypes of rs5743708 (*TLR2*) and rs5743551 (*TLR1*) loci, no statistically significant differences were found (p>0.05) (Table 1). A statistically significant correlation was established only for the rs4986790 polymorphism of the *TLR4* gene (p<0.05). However, despite the fact that the groups of patients with different genotypes of the rs4986790 (*TLR4*) locus differed statistically significantly (p<0.05) in terms of NET level, their pairwise comparison did not reveal a statistically significant correlation (Figure 2).

**Table 1:** Results of the analysis of correlation between rs5743551 (*TLR1*), rs5743708 (*TLR2*), and rs4986790 (*TLR4*) polymorphisms and NET level (Me [Q1; Q3])

Locus	Genotype	First blood sampling (n=38)		Second blood sampling (n=35)	
		n	NET level	n	NET level
rs5743551(*TLR* *1*)	AA	22	6.25 [4.40; 11.0]	22	2.80 [0.33; 6.60]
	AG	12	5.25 [3.10; 13.20]	9	2.0 [1.3; 6.4]
	GG	4	6.40 [5.70; 7.95]	4	2.75 [1.73; 4.50]
p* (Kruskal–Wallis test)			0.84		1.0
rs5743708(*TLR* *2*)	GG	35	5.70 [3.95; 10.75]	33	2.6 [0.6; 6.4]
	AG	3	11.0 [8.20; 11.95]	2	4.0 [2.65; 5.35]
	AA	0	—	0	—
p* (Mann–Whitney test)			0.33		0.89
rs4986790(*TLR4*)	AA	30	6.8 [4.9; 11.3]	29	3.1 [1.9; 6.7]
	AG	6	5.0 [3.5; 5.7]	5	0 [0; 1.3]
	GG	2	1.8 [1.2; 2.3]	1	1.6 [1.2; 1.9]
p* (Kruskal–Wallis test)			0.027		0.09

* Statistical significance was calculated using 3×2 contingency tables. The second blood sampling was performed in 35 patients because of a lethal outcome in 3 people on the 5^th^–7^th^ day.

No statistically significant differences were obtained when conducting a comparative analysis of the NET level in the patients with different genotypes of all the three polymorphisms on the 5^th^–7^th^ day of hospitalization, (p>0.05) (see Table 1).

A general trend towards a decrease in NET level was observed during the course of pneumonia, which indicates a decrease in the NETosis activity of neutrophils (p<0.05).

**Figure 3:**
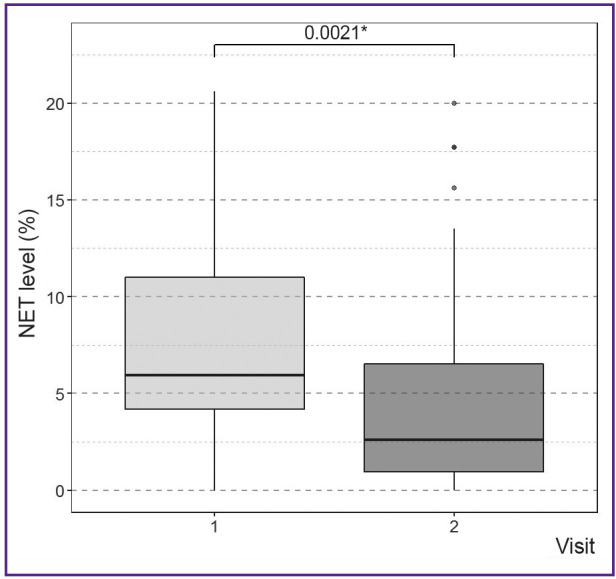
Dynamics of alteration in the NET level between the first and second blood sampling in patients * p-value of the nonparametric Wilcoxon test for paired samples

The overall decrease in NET level from the first day of blood sampling to the second blood sampling, without taking into account the genotype, was 32.1% on average (Figure 3).

The results of a comparative analysis of the genotype/ allele frequencies of the rs5743551 (*TLR1*), rs5743708 (*TLR2*), and rs4986790 (*TLR4*) loci with the level of NETs upon admission to hospital, i.e. at the time of the first blood sampling, are presented in Table 2.

**Table 2:** Comparative analysis of the genotype/allele frequencies of rs5743551 (*TLR1*), rs5743708 (*TLR2*), and rs4986790 (*TLR4*) loci in groups with low and high levels of NETs at the first blood sampling (n (%))

Locus	Genotype	NET level		p*
		Low (n=26)	High (n=12)	
rs5743551 (*TLR1*)	AA	15	7	1.0
	AG	8	4	
	GG	3	1	
	A allele	38 (73)	18 (75)	
	G allele	14 (27)	6 (25)	
rs5743708 (*TLR2*)	GG	25	10	0.233**
	AG	1	2	
	AA	0	0	
	G allele	51 (98)	22 (92)	
	A allele	1 (2)	2 (8)	
rs4986790 (*TLR4*)	AA	18	12	0.123
	AG	6	0	
	GG	2	0	
	A allele	42 (81)	24 (100)	0.026
	G allele	10 (19)	0	
	Dominant model (AA vs AG+GG)	18/8	12/0	0.039

* Statistical significance was calculated using Fisher’s test in contingency tables of 3×2 for genotypes and 2×2 for alleles; ** — a 2×2 contingency table for genotypes and alleles was used for the rs5743708 (*TLR2*) locus, due to the absence of one of the genotypes.

Statistically significant differences in the level of NETs were obtained only in relation to the rs4986790 polymorphism of the *TLR4* gene. The G allele is associated with a low level of NETs, the A allele — with a high level of NETs at the time of the first blood sampling in the patients (p=0.026). The analysis of the obtained data suggests a dominant model of the influence of the G allele in a patient’s genotype on the decrease in the NET level in his/her blood (p=0.039).

The comparative analysis of the dependence of NET level at the time of the first blood sampling on the frequencies of the genotypes and alleles of the rs5743551 (*TLR1*) and rs5743708 (*TLR2*) loci revealed no statistically significant differences between the groups of patients (p>0.05).

In order to identify the possible combined effect of several polymorphisms of the genes of the toll system, an additional analysis of the dependence of NET level on the combination of rare alleles of the studied polymorphisms in the genotype of the patients was carried out (Table 3).

**Table 3:** Frequency of rare allele combination in the rs5743551 (*TLR1*), rs5743708 (*TLR2*), and rs4986790 (*TLR4*) polymorphic loci in patients depending on the NET level in blood at the time of the first blood sampling

NET level	Presence of at least one rare allele of *TLR1*, *TLR2*, and *TLR4* gene polymorphisms	Combination of at least two rare alleles of *TLR1*, *TLR2*, and *TLR4* gene polymorphisms in one patient
Low (n=26)	16 (61.5%)	4 (15.4%)
High (n=12)	6 (50%)	1 (8.3%)
p (Fisher’s exact test)	0.725	1.0

There was a tendency towards the prevalence in the group of the patients with a low level of NETs of the persons with a combination of at least two of the three studied polymorphisms of the *TLR1*, *TLR2*, and *TLR4* genes in the genotype (15.4% of patients with a low level of NETs vs 8.3% of patients with a high level of NETs (p>0.05).

A comparative analysis of the genotype/allele frequencies for the rs5743551 (*TLR1*), rs5743708 (*TLR2*), and rs4986790 (*TLR4*) polymorphisms was carried out depending on the outcome of pneumonia (recovery/death) (Table 4).

**Table 4:** Comparative analysis of the genotype/allele frequencies for rs5743551 (*TLR1*), rs5743708 (*TLR2*), and rs4986790 (*TLR4*) polymorphisms in the groups of patients with different outcomes of pneumonia

Locus	Genotype	Outcome		p*	OR (95% CI)
		Favorable (n=31)	Lethal (n=7)		
rs5743551 (*TLR1*)	AA	22	0	<0.0001**	—
	AG	9	3		
	GG	0	4		
	А allele	53	3	<0.0001***	20.3 (4.3–135.0)
	G allele	9	11		
rs5743708 (*TLR2*)	GG	29	6	0.47	—
	AG	2	1		
	AA	0	0		
rs4986790 (*TLR4*)	AA	26	4	0.16	—
	AG	4	2		
	GG	1	1		
	A allele	56	10	0.08	3.65 (0.64–18.90)
	G allele	6	4		

* Statistical significance was calculated using Fisher’s test in contingency tables of 3×2 for genotypes and 2×2 for alleles; ** — 1.74·10^–^^5^; *** — 7.06·10^–^^6^.

It was found that the presence of the G allele in the genotype of the rs5743551 (*TLR1*) locus in a patient increases the risk of unfavorable outcome of the disease (p<0.0001) (OR=20.3; 95% CI (4.3–135.0).

However, to obtain more reliable statistical data, it is necessary to conduct a comparative analysis on a larger sample size.

It can be supposed that a type of a clinically significant bronchopulmonary pathogen has an impact on the level of NETs taking into consideration the data on lower level of NETs in the patients with AG and GG genotypes at the rs4986790 (*TLR4*) locus, and the results of the studies [8, 10], indicating that pathogenic microorganisms have different ways of protection against NETs, the relationship of *TLR4* gene polymorphism (rs4986790) with increased susceptibility to pneumococcal infection, as well as the presence of association between rs4986790 (*TLR4*) and the frequency of gram-negative flora detection in patients with severe and extremely severe pneumonia [19, 20]. However, the comparative intergroup analysis of the data obtained from the microbiological study of tracheobronchial aspirate and blood with different NET levels did not show statistically significant differences.

## Discussion

The presented study has revealed the role of NETs as a marker of the dynamics of the inflammatory process in patients with community-acquired pneumonia, which corresponds with the published data [21]. However, no data has been obtained indicating that, in patients with community-acquired pneumonia hospitalized in the ICU, NET level in smears made from venous blood is associated with the presence of inotropic support, risk factors for an unfavorable outcome, the development of complications, the outcome of the disease, the level of leukocytes and CRP, as well as with the type of a clinically significant causative agent of pneumonia, although according to the literature, we have found studies that demonstrate the presence of this relationship [22–26].

Thus, in the study published in 2019, which included 73 patients with sepsis of various etiology, it was found that the level of NETs in the group of survivors and deceased was statistically significantly different, and all the patients with NET concentrations of more than 23% died [27]. Our results can have the following explanations. The first reason is the insufficient severity of the systemic inflammatory response in the patients in the recruited group. Only 3 patients with pneumonia complications had sepsis, 9 of 38 patients were assigned to the 4^th^ class on the PORT scale, there were no patients of the 5^th^, most severe, class. The second reason relates to *Streptococcus pneumoniae* which is the leading etiological factor in the development of community-acquired pneumonia. In our study, it was the causative agent in 39% of patients, which led to a low variability of the sample for the etiological factor and made it difficult to conduct a comparative analysis. In addition, mortality in the patients with pneumonia was significantly lower than in the study by Gur’ev et al. [27] and amounted to 18.4% (7 out of 38). Probably, for this reason, we did not get a statistically significant difference between the groups of survived and deceased patients.

The absence of association between the nature of the pathogen and level of NETs in the presented study can be explained by the presence of defense mechanisms against neutrophil traps identified in pathogens of bronchopulmonary infection and discussed in the literature [21–23]. However, the method for determining NET level [15] used in our study takes into account only the ability of activated and circulating neutrophils to form NETs in a blood smear when they die. It is for this reason that microbial factors that destroy NETs do not affect the result of the analysis in any way, and the data obtained are objective.

We have established a negative relationship of medium strength between the frequency of occurrence of the genotypes of the *TLR1* and *TLR4* gene polymorphisms, their combinations, and level of NETs. A positive relationship of medium strength of the death rate and the presence of the AG and GG genotypes in rs5743551 (*TLR1*) and rs4986790 (*TLR4*) genes has also been found, respectively. A strong relationship has been established at the combination of these polymorphisms. Probably, the obtained data can be explained by the fact that a low level of neutrophil activity associated with the presence of the AG genotype of rs5743551 (*TLR1*) and GG genotype of rs4986790 (*TLR4*) is a marker of a violation of the genetically determined mechanism of innate immunity and can be a predictor of the lethal outcome of pneumonia.

This assumption is supported by the data obtained by other researchers. Hoogerwerf et al. [28] studied the effect of two groups of ligand-causative agents of respiratory infections on *TLRs*: the *TLR2* ligand (a component of gram-positive bacteria) and the *TLR4* ligand (a component of gram-negative bacteria). The authors suggested that *TLR2* or *TLR4* stimulation leads to various pathomorphological variants of the inflammatory process in the lung tissue.

A study conducted by Siebert et al. [10] showed that a decreased level of *TLR4* expression may be an additional factor underlying susceptibility to pneumococcal infection. Skerrett et al. [29] also demonstrated the contribution of *TLR2* to respiratory protection against bacterial infection.

## Conclusion

In the conducted study, the influence of the rs4986790 polymorphism of the *TLR4* gene on the level of NETs was observed in patients with community-acquired pneumonia on the 1^st^ or 2^nd^ day of hospitalization. The AG and GG genotypes for the given locus of the *TLR4* gene were associated with a lower level of NETs compared with non-carriers of polymorphism. In the present study, the G allele for the rs5743551 polymorphism of the *TLR1* gene was associated with a poor outcome of the disease. The data obtained suggest that NETs are markers of neutrophil activity, which closely correlated with the studied genetic polymorphisms and influence the prognosis of the pneumonia outcome. Further investigation of the effect of *TLR1* and *TLR4* polymorphisms in the immunological response in pneumonia is needed.
